# Supplementary material for: Genome editing abrogates angiogenesis in vivo
Source: Nat Commun. 2017 Jul 24;8:112. doi: 10.1038/s41467-017-00140-3 (PMC5524639; doi:10.1038/s41467-017-00140-3)
Supplement: Supplementary file 1 — Supplementary Information [file 41467_2017_140_MOESM1_ESM.pdf]

File Name: Supplementary Information
Description: Supplementary Figures.

File Name: Peer Review File
Description:

Supplementary Information
Supplementary Figures

Supplementary Figure 1

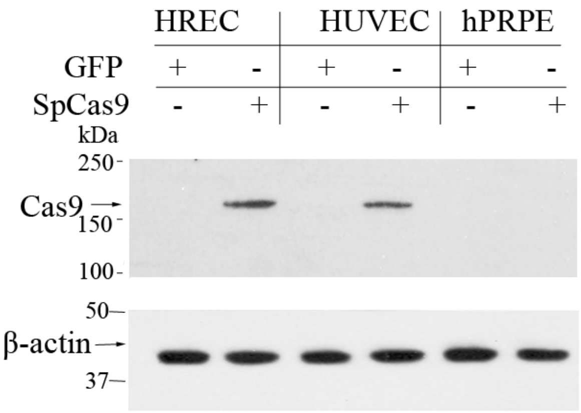

pICAM2-driven expression of SpCas9 in ECs. As described in Figure 1, after transduction with rAAV1-CMV-GFP (GFP) or rAAV1-pICAM2-SpCas9 (SpCas9) (2μl/well, 3.75 x10^{12}vg/ml) in a 48-well plate for four days, cell lysates were subjected to western blot analysis with antibodies against Cas9 and β-actin. Data shown are representative of three independent experiments. kDa:kilodalton. HREC: human retinal microvascular endothelial cells, HUVEC: human umbilical vein cells, hPRPE: human primary retinal pigment epithelial cells.

Supplementary Figure 2

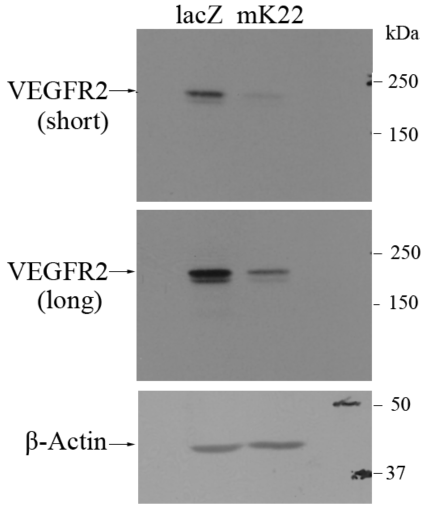

Depletion of VEGFR2 expression using AAV-CRISPR/Cas9. Total cell lysates from the transduced MVECs (mouse primary brain vascular endothelial cells) were subjected to western blot analysis with antibodies against VEGFR2 (short: short exposure, long: long exposure) and β-actin. Data shown are representative of three independent experiments. lacZ: lacZ-sgRNA as a negative control, mK22: VEGFR2-sgRNA.

Supplementary Figure 3

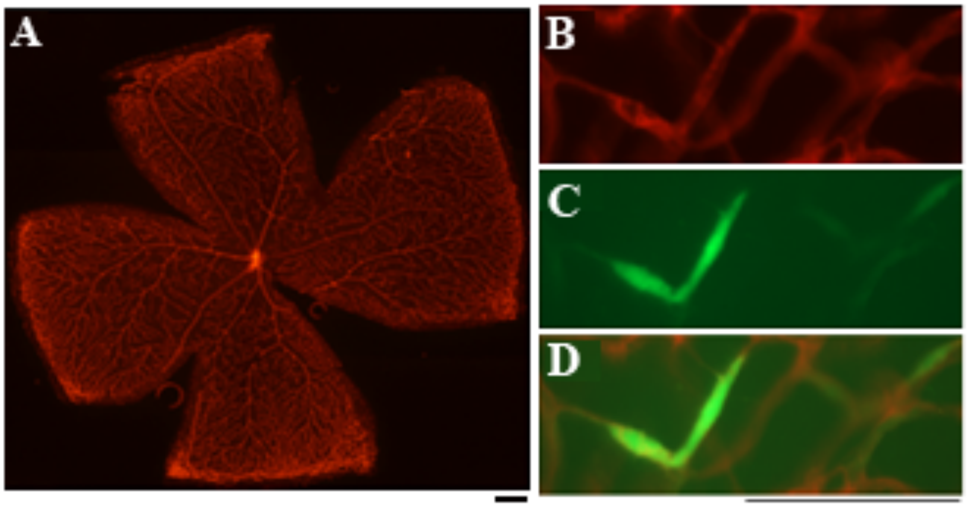

rAAV1 transduction of ECs in the normal retinal vessel. On P12, control mice (raised in room air) were intravitreally injected with rAAV1 with a GFP gene driven by CMV) (rAAV1-CMV-GFP) (1 μl, 3.75 × 10^{12} vg/ml) from the Gene Transfer Vector Core at Schepens Eye Research Institute (Boston, MA). On P17, after euthanasia, the mouse eyes were carefully removed and fixed in 3.7% paraformaldehyde. Retinas were dissected, whole-mounted and then stained overnight at 4 °C with murine-specific EC marker isolectin 4 (IB4)-Alexa 594 (red) [23, 38-39]. The images were taken with an EVOS FL Auto microscope. (**A**) Normal superficial retinal vessels from a control mouse, scale bar: 500 μm; (**B-D**) Partial superficial retinal vessel from one of the rAAV1-CMV-GFP injected mice raised in room air. (**B**) IB4 staining of ECs (TxRed channel), (**C**) GFP expression (GFP channel), (**D**) A merged image of **B** & **C**, Scale bar: 200 μm. Each figure represents six ones from different mice.

Supplementary figure 4

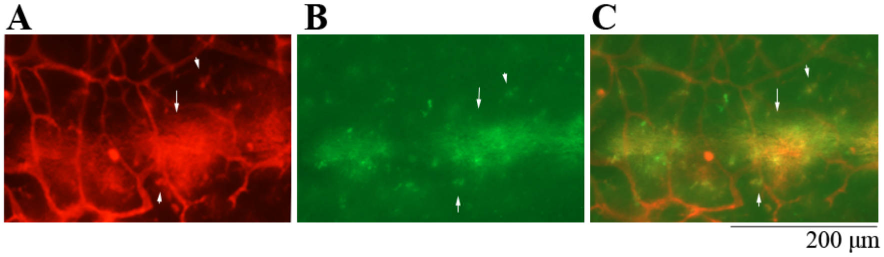

rAAV1 transduction of ECs in the pathological retinal capillaries. On P12, experimental of mice (raised in 75% oxygen for five days) were intravitreally injected with rAAV1-CMV-GFP (1 μl, 3.75 × 10^{12} vg/ml). On P17, after euthanasia, the mouse eyes were carefully removed and processed as for supplementary figure 1 [23, 38-39]. The images represent six ones from different mice and were taken with an EVOS FL Auto microscope.

Supplementary Figure 5

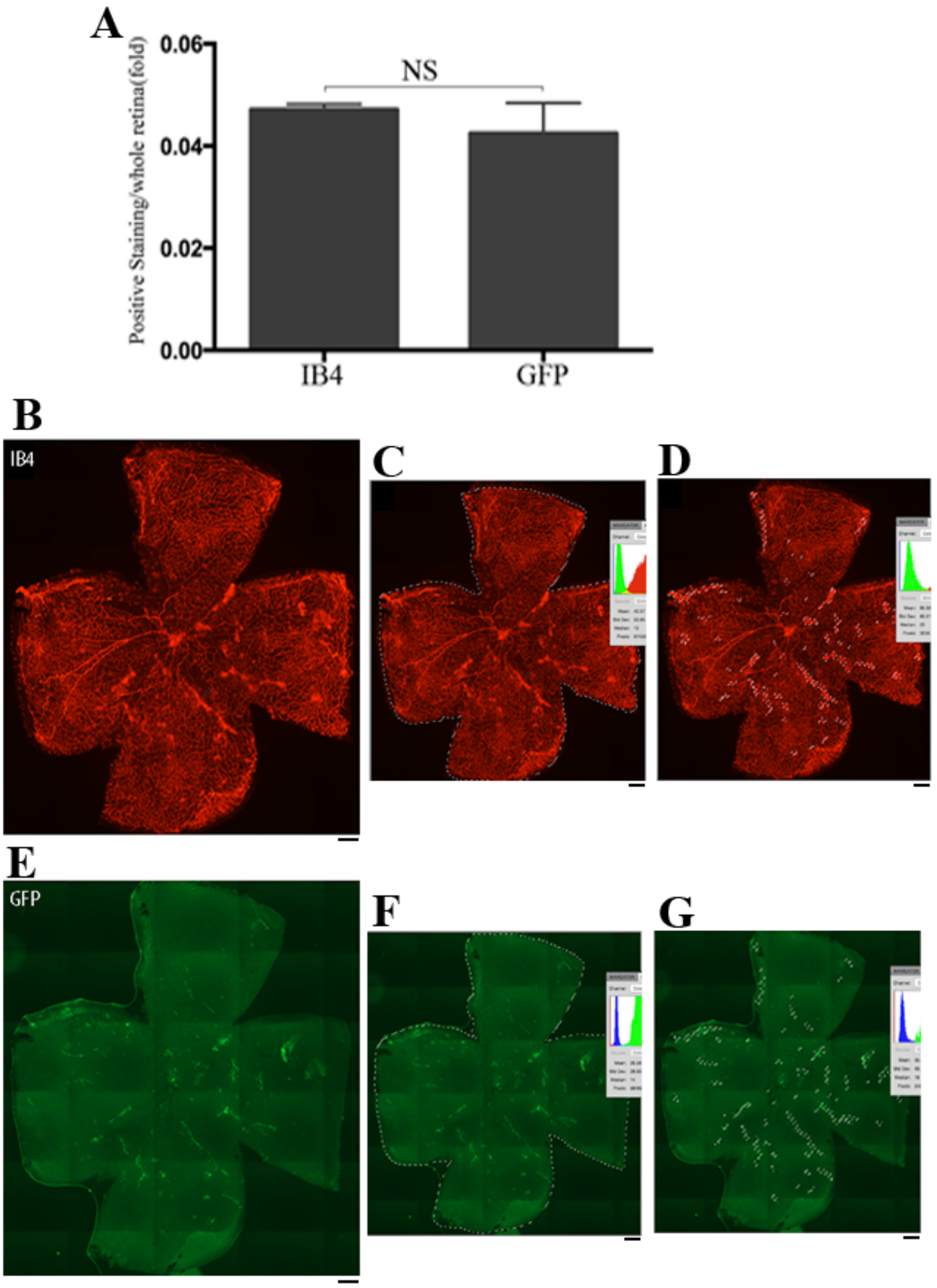

Analysis of transduced ECs and pathological retinal vessel. A. The pixels of IB4 staining in pathological vessels (IB4) or GFP positive stain (GFP) to those of the whole retinal area were folded. There was no significant difference between the quantitation of IB4 staining in pathological vessels and GFP positive staining (n =6). NS: No significant difference. Screen shots of the whole retina IB4 or GFP staining when quantitation was

performed as described in Methods and previously[3]. Briefly, quantification of neovascularization (NV) at P17. (**B-D**) Images of retinal whole-mount stained for endothelial cells with isolectin B4-594; (**E-G**) Images of retinal whole-mount for GFP under green channel. Scale bar: 500 μm.

Supplementary figure 6

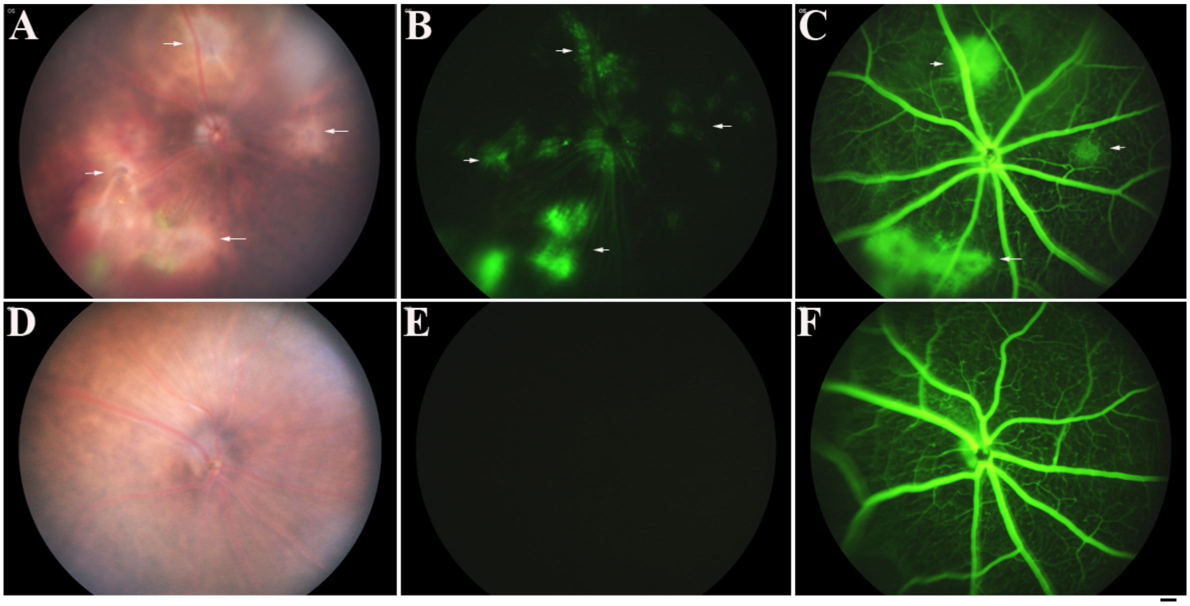

rAAV1 infection of the laser-injured area. After laser photocoagulation of the eyes of eight week-old mice (C57BL/6J) using a Streampix5 laser system, rAAV1 (1 μl, 3.75×10^{12} vg/ml) was injected into the vitreous of the left eye (A, B, and C). The right one (D, E and F) was uninjected and served as a control. On day seven, fundus images were taken using the Micron III retina imaging system with illumine (A, D) or UV light (B, E). Then 0.01 ml of 25% sodium fluorescein was injected intraperitoneally. Images of fluorescein angiography represent six ones from different mice and were taken with UV light (C, F). Scale bar: 500 μm.

Supplementary figure 7

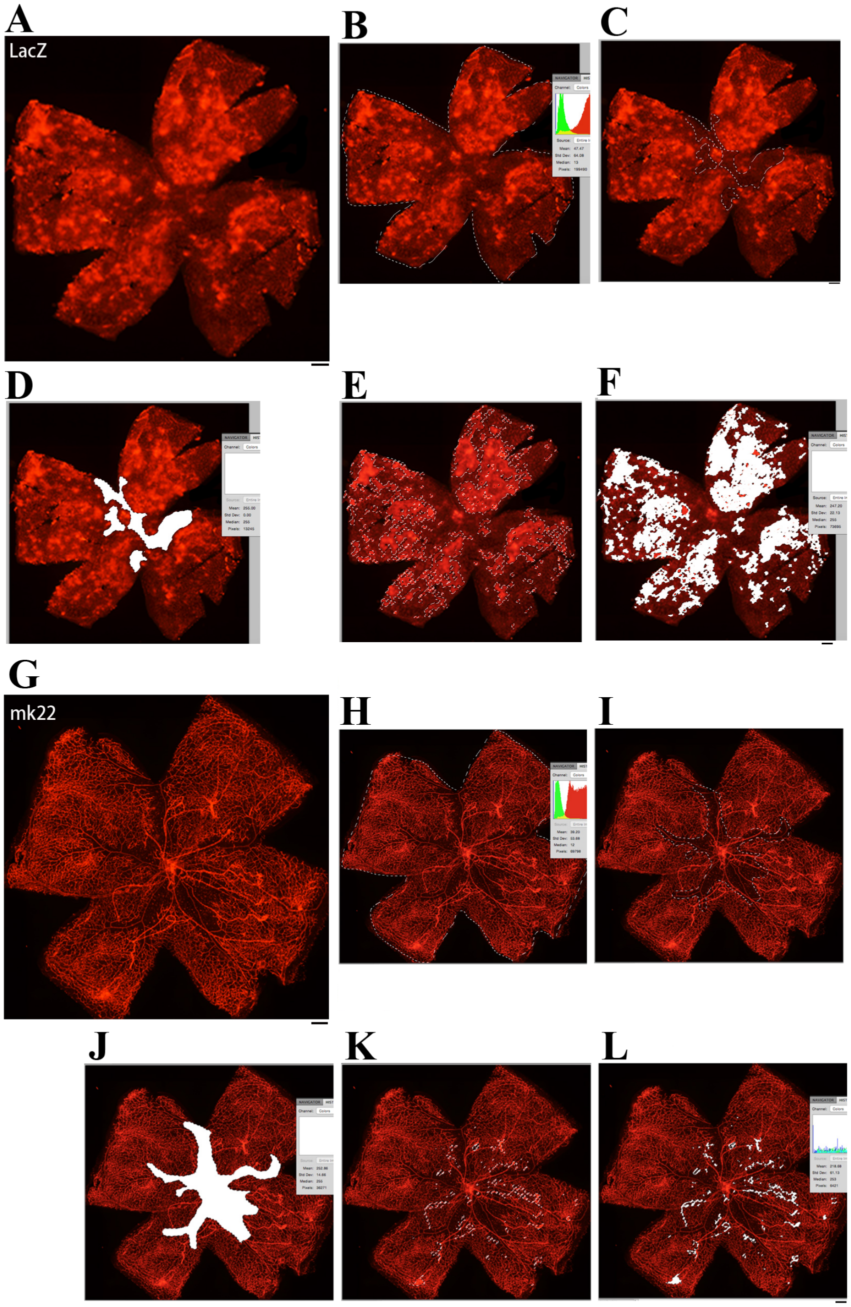

Analysis of vaso-obliteration and NV area at P17 (n = 6) conducted as described in Methods and previously[3]. **A.** Image of retinal whole-mount stained for endothelial cells with isolectin B4-594 from a mouse intravitreally injected with AAV-SpCas9 and AAV-*lacZ*-sgRNA (mK22); (**B-D**) retinal whole-mount with entire avascular (vaso-obliterated) area highlighted in white. (**E-F**) Screenshots of retinal whole-mount with the neovascular tufts highlighted. **G.** Image of retinal whole-mount stained for endothelial cells with isolectin B4-594 from a mouse intravitreally injected with AAV-SpCas9 and AAV-*VEGFR2*-sgRNA (mK22); (**H-J**) retinal whole-mount with entire avascular (vaso-

obliterated) area highlighted in white. (**K-L**) Screenshots of retinal whole-mount with the neovascular tufts highlighted. Scale bar: 500 μm.

Supplementary Figure 8

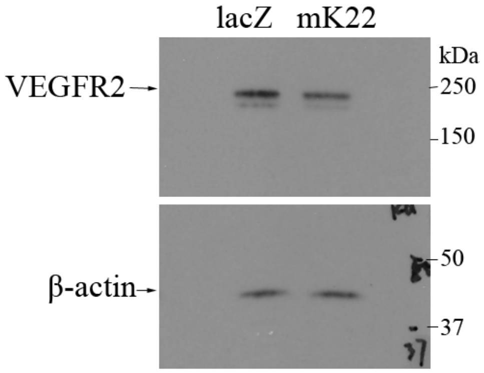

Depletion of VEGFR2 in retinas. The lysates of the rAAV1-SpCas9/lacZ –sgRNA (lacZ) or –VEGFR2-sgRNA (mK22) -injected retinas were subjected to western blot analysis using indicated antibodies. This is representative of three independent experiments.

Supplementary figure 9

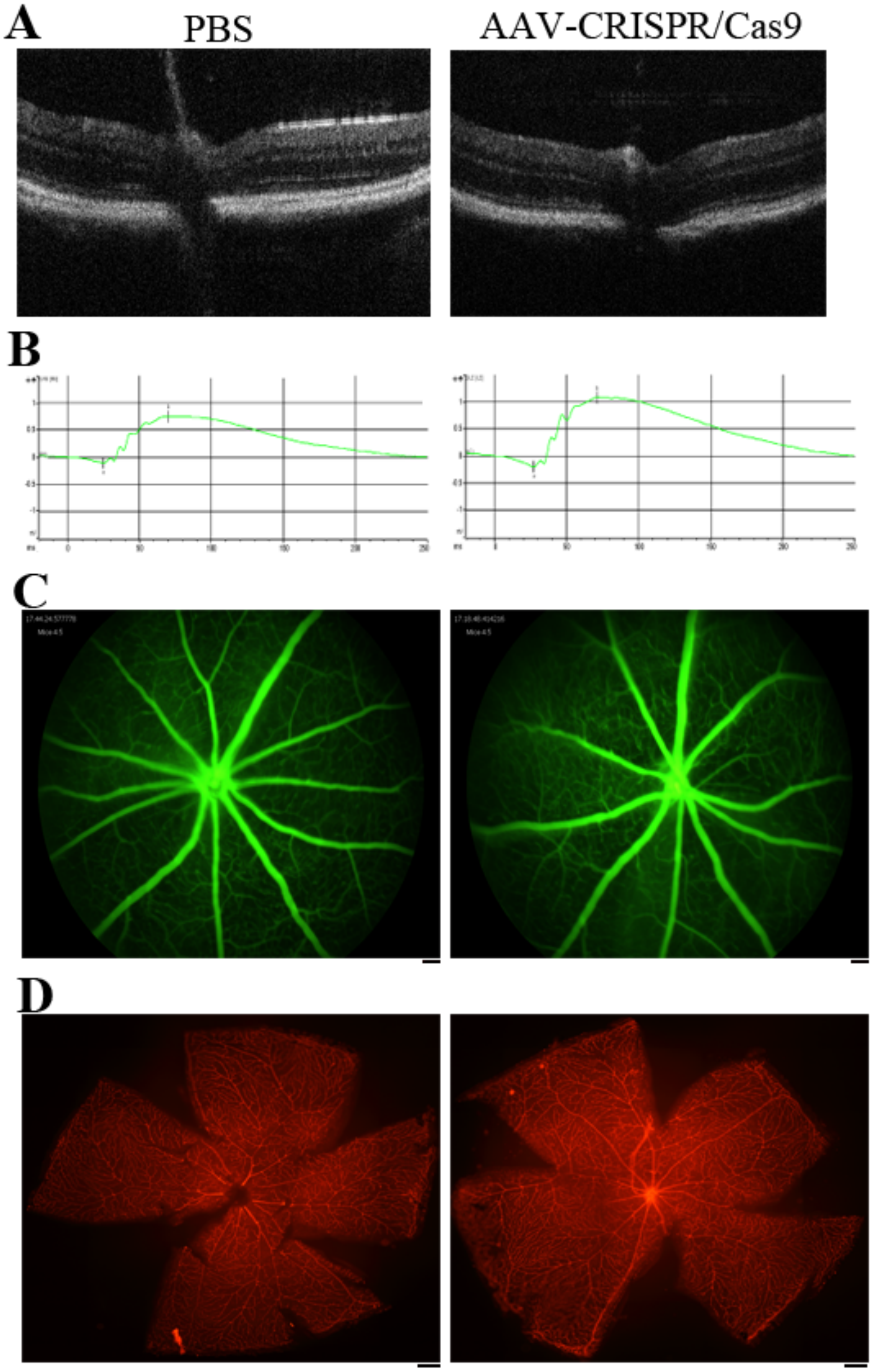

Examination of toxicity of the dual AAV-CRISPR/Cas9. Five P12 mice were injected with the dual AAV-CRISPR/Cas9 (1 μl, 3.75×10^{12} vg/ml) or 1 μl of phosphate buffered saline (PBS) into the left eye or the right eye, respectively. After four weeks, the mice were examined by optical coherence tomography (OCT) (A), electroretinography (ERG) (B), fluorescein fundus angiography (FFA) (C) and whole-mount retinal staining with IB4 (D). Scale bar: 500 μm.
